# Aging in Schizophrenia: Clinical, Biological, and Psychosocial Perspectives

**DOI:** 10.3390/geriatrics11050133

**Published:** 2026-09-16

**Authors:** Constanza Morén, Alonso Pérez-Toribio, Enric Rubio-Contreras, Patricia Álvaro, Antonio Rosa, Sara Pedregosa

**Affiliations:** 1 Fundamental and Clinical Nursing Department, Nursing Faculty, University of Barcelona, L’Hospitalet de Llobregat, 08907 Barcelona, Spain; alonperez11@ub.edu (A.P.-T.); sarapedregosa@ub.edu (S.P.); 2 August Pi i Sunyer Biomedical Research Institute (IDIBAPS), 08036 Barcelona, Spain; enrubio@recerca.clinic.cat; 3 Clínic Foundation for Biomedical Research (FCRB), 08036 Barcelona, Spain; 4 Centro de Salud Mental CSMA, Fundació CPB Serveis Salut Mental, Dreta de l’Eixample, 08025 Barcelona, Spain; palvaro@cpbssm.org

**Keywords:** schizophrenia, aging, comorbidity, cognitive decline, psychosocial factors

## Abstract

**Background/Objectives**: Schizophrenia is a chronic psychiatric disorder traditionally associated with early adulthood; however, an increasing number of individuals are now reaching older age, highlighting the need to better understand the aging process in this population. Emerging evidence suggests that schizophrenia may be associated with accelerated aging, characterized by an earlier onset of age-related conditions and increased vulnerability to physical and cognitive decline. **Methods**: We provide an integrative overview of aging in schizophrenia, focusing on biological mechanisms, clinical manifestations, and psychosocial dimensions. **Results**: Key biological processes, including oxidative stress, mitochondrial dysfunction, chronic immune activation, and dysregulation of stress-response systems, are discussed as potential contributors to accelerated aging. Clinically, older individuals with schizophrenia present a high burden of cardiometabolic comorbidities, persistent cognitive impairment, and increased functional dependency, often compounded by the complexity of pharmacological management in this age group. In addition, psychosocial factors such as social isolation, stigma, and caregiver burden play a critical role in shaping health outcomes and quality of life. **Conclusions**: Despite growing recognition of these challenges, aging in schizophrenia remains an underexplored area, with significant gaps in longitudinal research and limited focus on older populations. A comprehensive approach that integrates biological, clinical, and psychosocial perspectives is essential to better characterize aging trajectories and to inform more effective and person-centered care strategies in this vulnerable population.

## 1. Introduction

Schizophrenia is a chronic and severe mental disorder that affects approximately 1% of the global population and is associated with substantial functional impairment, medical comorbidity, and reduced life expectancy [1]. Schizophrenia is considered one of the top ten causes of health burden in the World [2]. Although traditionally considered a disorder of early adulthood, advances in clinical care and social support have led to an increasing number of individuals with schizophrenia reaching older age [3]. This demographic shift has brought renewed attention to the complex interplay between aging and schizophrenia, a field that remains insufficiently explored.

Individuals with schizophrenia experience a markedly reduced life expectancy, estimated to be 15–20 years shorter than that of the general population [4], largely driven by cardiovascular disease, metabolic disorders, lifestyle-related factors, and suicide, among others [5]. Beyond this, accumulating evidence suggests that schizophrenia may be associated with processes of accelerated biological aging. Alterations in oxidative stress pathways, chronic low-grade inflammation [6], mitochondrial dysfunction [7,8], and dysregulation of stress-response systems have all been implicated, potentially contributing to earlier onset of age-related conditions and increased frailty.

From a clinical perspective, aging in schizophrenia is characterized by a high burden of physical comorbidities, persistent cognitive deficits, and progressive functional decline [9]. Distinguishing between long-standing cognitive impairment and superimposed neurodegenerative processes remains a significant challenge in older patients. Furthermore, pharmacological management becomes increasingly complex with advancing age due to changes in pharmacokinetics, increased sensitivity to adverse effects, and the frequent presence of polypharmacy [10]. Moreover, comorbid alcohol and substance use disorders, sometimes referred to as “dual pathology” when co-occurring with schizophrenia [11], are also common and may further contribute to poorer physical health, treatment outcomes, and overall prognosis [12]. The cumulative impact of these factors may become particularly relevant with advancing age.

In addition to biological and clinical factors, psychosocial dimensions play a crucial role in shaping outcomes in older adults with schizophrenia. Social isolation, stigma, and caregiver burden are highly prevalent and often intensify with age [13], highlighting the need for integrated models of care that extend beyond symptom control.

Despite these challenges, aging in schizophrenia remains an under-researched area, with limited integration between psychiatric, geriatric, and translational research frameworks. A deep understanding of this topic requires bridging biological mechanisms with clinical manifestations and lived experience [14]. An integrative perspective is therefore needed to understand how these interconnected dimensions jointly shape aging trajectories in schizophrenia and to translate this knowledge into appropriate care for an increasing population of older adults with the disorder.

Accordingly, the aim of this comprehensive review is to synthesize current evidence on aging in schizophrenia across biological, clinical, pharmacological, and psychosocial domains, integrating these dimensions within a unified framework of accelerated aging. By bridging traditionally separated areas of research, this review seeks to identify key challenges and knowledge gaps and to inform more effective, person-centered approaches to care in this growing population.

A narrative literature review was conducted using the PubMed database to identify relevant studies (period 2000–2026). Relevant articles were identified using combinations of keywords related to schizophrenia, aging, comorbidity, cognition, pharmacological treatment, and psychosocial factors in older adults. The search strategy included terms such as “schizophrenia”, “aging”, “accelerated aging”, “mitochondrial dysfunction”, “oxidative stress”, “HPA axis”, “older adults”, “comorbidity”, “cognitive decline”, “antipsychotics”, and “psychosocial factors”, combined using Boolean operators (AND, OR). For each dimension, domain-specific search terms were combined with “schizophrenia” AND “aging” (e.g., biological terms AND schizophrenia AND aging; clinical terms AND schizophrenia AND aging; psychosocial terms AND schizophrenia AND aging). Within each domain, additional terms relevant to the specific topics covered in the review were used to refine the searches. Titles and abstracts were screened for relevance, and full-text articles were reviewed to extract key findings. Additional references were identified through the bibliographies of selected articles. Given the narrative design of this review, no formal quality assessment or meta-analysis was performed. Studies were included if they focused on schizophrenia or schizophrenia spectrum disorders and addressed aspects related to aging, including biological mechanisms, clinical outcomes, or psychosocial factors relevant to aging. Both original research articles and relevant review papers were considered. Studies were excluded if they focused on psychiatric disorders other than schizophrenia, unless results were clearly stratified for schizophrenia. Articles not related to aging processes, not available in full text, or not written in English were also excluded.

Given the narrative and integrative nature of this review, the literature search was not intended to provide an exhaustive systematic identification of all available studies, and no formal risk-of-bias assessment or meta-analysis was performed. Rather, the literature was selected to provide a broad and clinically meaningful synthesis of the biological, clinical, and psychosocial dimensions of aging in schizophrenia.

## 2. Aging in Schizophrenia: Conceptual Framework

In this review, “older adults” generally refers to individuals aged 65 years and above, in accordance with the conventional threshold commonly used in geriatric and old-age psychiatry. However, this chronological threshold should be interpreted cautiously in schizophrenia. Evidence of accelerated biological and clinical aging suggests that age-related vulnerabilities, multimorbidity, functional impairment, and other geriatric-like features may emerge at younger chronological ages in this population. Therefore, the present review also considers evidence from middle-aged adults when it provides relevant information on aging trajectories or the early emergence of age-related outcomes. This distinction is particularly relevant for healthcare organizations, as individuals with schizophrenia may develop aging-related care needs before reaching the conventional age threshold for transition from adult to old-age services.

Aging is a complex and multifactorial process involving progressive biological, functional, and psychosocial changes over time [15]. In the general population, aging is typically characterized by gradual physiological decline and increased vulnerability to chronic diseases. However, in individuals with schizophrenia, this process appears to follow a distinct trajectory, often described as accelerated or premature aging [16].

The concept of accelerated aging in schizophrenia refers to the observation that individuals with this disorder tend to develop age-related conditions earlier and experience a higher burden of morbidity than the general population [3]. This phenomenon is supported by epidemiological data showing increased rates of cardiovascular disease, metabolic disorders [17], and frailty at younger ages. In addition, biological studies have identified alterations in molecular pathways commonly associated with aging, including oxidative stress [18], chronic neuroimmune activity, mitochondrial dysfunction [7], and dysregulation of stress-response systems [19].

One key framework for understanding this process is the concept of allostatic load, which describes the cumulative physiological burden resulting from chronic exposure to stress [20]. In schizophrenia, persistent activation of stress-related systems—particularly the hypothalamic–pituitary–adrenal (HPA) axis—may contribute to long-term dysregulation across multiple biological systems [21]. Over time, this sustained burden can lead to multisystem impairment, thereby accelerating biological aging and increasing vulnerability to both physical and mental health deterioration. Importantly, accelerated aging in schizophrenia is not solely driven by intrinsic biological mechanisms. Lifestyle factors such as smoking [22], poor diet [23], physical inactivity [24], and limited access to healthcare play a significant role. Social determinants, including stigma, isolation, and socioeconomic disadvantage, further compound these effects, creating a complex interplay between biological and environmental factors.

Despite growing recognition of accelerated aging in schizophrenia, the concept remains heterogeneous and incompletely understood. Distinguishing between normal aging, disease-related progression, and treatment-related effects represents a major challenge in both research and clinical practice. A comprehensive approach that integrates biological, clinical, and psychosocial dimensions is therefore essential to better characterize aging trajectories in this population.

## 3. Biological Factors

### 3.1. Biological Mechanisms Underlying Accelerated Aging in Schizophrenia

Schizophrenia is accompanied by accelerated biological aging, already by mid-adulthood [25], as supported by evidence from systemic biomarkers and other biological measures across multiple physiological systems [26], including oxidative, metabolic, genetic and synaptic factors, among others. Moreover, recent single-nucleus transcriptomic analyses of the human prefrontal cortex identified a coordinated neuron–astrocyte gene-expression program that declines both with advancing age and in schizophrenia, suggesting shared molecular features between schizophrenia and brain aging [27].

Although no single mechanism fully explains this phenomenon, a combination of interconnected processes—including oxidative stress [18], mitochondrial dysfunction [8], immune dysregulation [6], and alterations of stress-response pathways [21]—appears to contribute to the progressive decline observed in these patients. Measures of biological aging could prove valuable for assessing patients’ risk for physical and cognitive decline and for evaluating intervention effectiveness [25].

#### 3.1.1. Mitochondrial Dysfunction

Mitochondrial dysfunction represents another key mechanism linking schizophrenia and aging. Mitochondria play a central role in cellular energy production through oxidative phosphorylation (OXPHOS), and their impairment can lead to bioenergetic deficits, increased oxidative stress, and activation of apoptotic pathways [28]. Alterations in mitochondrial function have been consistently reported in schizophrenia across different models, including peripheral tissues and neuronal systems [29]. These alterations may contribute not only to neurobiological dysfunction but also to systemic manifestations such as fatigue [30], metabolic dysregulation [17], and reduced physiological resilience, as impaired mitochondrial energy production can reduce cellular ATP availability, while mitochondrial dysfunction may also increase reactive oxygen species (ROS) production and disrupt metabolic homeostasis. Together, these processes may compromise the ability of cells and tissues to adapt to physiological stress, thereby contributing to these systemic manifestations.

#### 3.1.2. Mitochondrial-Related Oxidative Stress

Mitochondrial dysfunction can lead to increased production of ROS and, consequently, oxidative stress [28]. Oxidative stress has been widely implicated in the pathophysiology of schizophrenia [31] and is also a well-established hallmark of aging [32]. An imbalance between the production of ROS and antioxidant defense systems, particularly involving glutathione, may lead to cumulative cellular damage, including oxidative modifications of lipids, proteins, and DNA, which may progressively impair cellular structure and function [33]. This imbalance is especially relevant in the brain, where high metabolic demand and lipid-rich environments increase vulnerability to oxidative injury [34]. In schizophrenia, reduced antioxidant capacity has been associated with neuronal dysfunction, particularly affecting interneurons involved in cognitive processing [35].

#### 3.1.3. Age-Related Chronic Immune Activation

Chronic low-grade immune activation is increasingly recognized as a shared pathway between schizophrenia and aging [36]. Elevated levels of pro-inflammatory cytokines, such as interleukin-6 and tumor necrosis factor-alpha, have been observed in patients with schizophrenia [37] and may contribute to neurotoxicity, synaptic dysfunction, and altered neurodevelopmental trajectories. These immune alterations should not necessarily be attributed exclusively to the disorder itself. Antipsychotic treatment may also modulate immune signaling, and some agents, including clozapine, have been associated with changes in circulating pro-inflammatory cytokines. Thus, the immune profile observed in schizophrenia may reflect a complex interplay between disease-related processes and treatment-related effects, among other potential contributing factors. Over time, this persistent inflammatory state may exacerbate vulnerability to age-related diseases, including cardiovascular and neurodegenerative conditions [38]. These patterns resemble geriatric syndromes typically observed in advanced age, but which appear earlier in schizophrenia [39], including frailty, cognitive impairment, functional decline, and increased vulnerability to falls.

Importantly, although the term “neuroinflammation” is frequently used in the context of schizophrenia, its application remains controversial. Recent perspectives argue that this term may be misleading, as current evidence does not consistently support the presence of a classical inflammatory process in the brain, as observed in neurological or autoimmune conditions [40]. Instead, findings in schizophrenia are more accurately described as subtle and heterogeneous alterations involving both quantitative changes in inflammatory mediators and potentially qualitative differences in immune responses, rather than as a uniform and well-defined inflammatory state. This distinction is relevant not only from a mechanistic standpoint but also for the interpretation of biomarker studies and the development of targeted interventions. Importantly, the magnitude and direction of these alterations may vary across patients, disease stages, treatments, and biological compartments, further supporting the concept of immune dysregulation rather than a single classical inflammatory process.

Overall, immune dysregulation in schizophrenia appears to reflect a complex, systemic process that interacts with other biological mechanisms of aging, including oxidative stress, mitochondrial dysfunction, and neuroendocrine alterations, contributing to a cumulative physiological burden over time. These processes are closely interconnected, as altered immune signaling can promote ROS production and oxidative stress, which may impair mitochondrial function, while stress-related neuroendocrine pathways, particularly the HPA axis, can in turn modulate immune responses, creating mutually reinforcing mechanisms of cellular and physiological dysregulation.

#### 3.1.4. Stress HPA Axis and Epigenetic Clocks in Schizophrenia

In addition, dysregulation of the hypothalamic–pituitary–adrenal (HPA) axis may further contribute to biological aging in schizophrenia [41]. Prolonged exposure to elevated cortisol levels can have widespread effects, including metabolic alterations, immune suppression, and structural brain changes. This endocrine imbalance may interact with oxidative and inflammatory pathways, reinforcing a cycle of cumulative physiological burden.

Importantly, these biological mechanisms do not operate in isolation but rather interact dynamically, contributing to a multisystem process of accelerated aging. In this context, genetics and epigenetics also play a role. On one hand, the trajectory of gene expression changes associated with brain aging differs between individuals with schizophrenia and unaffected controls, suggesting a disruption of normal age-related transcriptional processes in schizophrenia [42]. On the other hand, epigenetic clocks have emerged as promising biomarkers of biological aging [43]. These clocks are based on DNA methylation patterns across specific genomic sites and provide an estimate of an individual’s “biological age”, which may differ from chronological age. Studies using epigenetic clocks have provided evidence of altered biological aging in schizophrenia, although findings regarding age acceleration vary according to the specific clock, age range, and population examined. Thus, these measures may provide an integrative readout of cumulative biological stress and age-related physiological changes. Understanding these interconnected pathways is essential to identify potential therapeutic targets and to develop strategies aimed at mitigating long-term disease burden in individuals with schizophrenia.

## 4. Clinical Factors

### 4.1. Physical Health and Comorbidities in Aging Individuals with Schizophrenia

Individuals with schizophrenia exhibit a significantly higher burden of physical comorbidities than the general population [44], which contributes substantially to reduced life expectancy and poorer overall health outcomes. This burden becomes particularly pronounced with aging, reflecting the combined effects of disease-related factors, long-term treatment, and lifestyle influences.

Cardiometabolic disorders are among the most prevalent comorbidities in this population. Patients with schizophrenia show increased rates of obesity, type 2 diabetes, dyslipidemia, and hypertension, all of which contribute to a markedly elevated risk of cardiovascular disease [45]. These alterations are influenced by multiple factors, including sedentary behavior [24], unhealthy dietary patterns [23], and high rates of smoking [22], which remain significantly more common in individuals with schizophrenia than in the general population.

Pharmacological treatment, particularly with antipsychotic medications, plays a relevant role in the development of metabolic disturbances. Antipsychotic treatment may contribute to weight gain, insulin resistance, lipid abnormalities, and other cardiometabolic disturbances, although the magnitude of these effects varies considerably across individual agents [46]. While these treatments are essential for symptom control, their long-term impact requires careful monitoring, especially in older adults.

In addition to cardiometabolic conditions, other chronic diseases such as respiratory disorders, infectious diseases, and musculoskeletal problems are also more prevalent in individuals with schizophrenia [47]. These conditions may be exacerbated by reduced access to healthcare, underdiagnosis, and lower adherence to medical treatments.

Aging further amplifies these challenges. Physiological changes associated with aging, such as decreased organ reserve and increased vulnerability to stressors, interact with pre-existing health conditions, leading to greater frailty and functional decline. Importantly, the concept of frailty—characterized by reduced resilience and increased risk of adverse outcomes—has gained attention as a relevant framework to understand aging in schizophrenia [39].

Despite the high burden of comorbidity, individuals with schizophrenia often receive suboptimal medical care [48,49]. Barriers such as stigma, fragmented healthcare systems, and difficulties in communication can lead to delayed diagnosis and inadequate management of physical illnesses. These challenges, together with the earlier emergence of age-related morbidity in schizophrenia, highlight the need for integrated care approaches that address both mental and physical health across the aging process. Particular attention to preventive healthcare and screening for age-related conditions may therefore be warranted at younger chronological ages in this population. However, preventive strategies should be guided by individual risk profiles rather than by assuming a fixed equivalence between chronological and biological age.

### 4.2. Cognitive Decline and Functional Outcomes

Cognitive impairment is a core feature of schizophrenia and represents one of the main determinants of long-term functional outcome [50]. Unlike other symptoms that may fluctuate over time, cognitive deficits are typically present from early stages of the disorder and tend to persist throughout the lifespan. With advancing age, the trajectory of cognitive decline in individuals with schizophrenia becomes increasingly complex, raising important questions regarding the interaction between disease-related processes and normal aging. As individuals with schizophrenia age, they often experience a progressive reduction in positive symptoms, while negative symptoms tend to persist and may even become more clinically prominent. Delusions, hallucinations, and behavioral disturbances generally diminish with age and may disappear in some cases. In contrast, negative symptoms such as avolition, apathy, blunted affect, poverty of speech, social withdrawal, disengagement from the environment, mutism, and negativism tend to persist in a chronic manner. Given the limited efficacy of antipsychotic medications in the treatment of negative symptoms, a gradual dose reduction may be considered a reasonable therapeutic strategy in older patients. However, this approach should be individualized, carefully balancing potential benefits against the risk of relapse, and always implemented under close clinical supervision.

In this context, from a biological perspective, alterations in neuroplasticity-related mechanisms have also been reported, including decreased levels of brain-derived neurotrophic factor (BDNF) in older individuals with schizophrenia, which have been associated with cognitive impairment [51].

In general, individuals with schizophrenia exhibit impairments across multiple cognitive domains, including attention, working memory, executive function, and processing speed [50]. These deficits are strongly associated with reduced functional capacity, affecting the ability to perform activities of daily living, maintain social relationships, and achieve independent living. Importantly, cognitive impairment in schizophrenia is often only partially responsive to pharmacological treatment, highlighting the need for complementary therapeutic strategies [52].

One of the major challenges in older adults with schizophrenia is distinguishing between long-standing cognitive deficits and superimposed neurodegenerative processes, such as dementia [53]. While individuals with schizophrenia may be at increased risk of developing dementia, cognitive impairment associated with long-standing schizophrenia should not be equated with a neurodegenerative process. Chronic schizophrenia and Alzheimer’s disease may share clinical manifestations, including memory impairment, social withdrawal, and psychomotor slowing, which can complicate differential diagnosis in older adults [54]. Neuroimaging approaches, particularly MRI and PET, may provide complementary information to help distinguish schizophrenia-related brain alterations from neurodegenerative processes characteristic of Alzheimer’s disease [54]. Cognitive decline in schizophrenia does not always follow the same trajectory as in neurodegenerative disorders, and in some cases may remain relatively stable over time. This variability complicates diagnosis and clinical management, particularly in advanced age.

Functional outcomes are closely linked to cognitive performance but are also influenced by social and environmental factors. Aging individuals with schizophrenia frequently experience progressive loss of autonomy, increased dependence, and higher rates of institutionalization. These outcomes are often exacerbated by comorbidities, reduced social support, and persistent stigma [55]. In fact, a double social stigma exists in relation to schizophrenia and aging [55].

The concept of functional aging has therefore emerged as a relevant framework, emphasizing not only cognitive decline but also the broader capacity to adapt to age-related changes [15]. Interventions targeting cognitive function, including cognitive remediation therapies [56] and psychosocial support [57], may play a crucial role in preserving independence and improving quality of life in this population.

Importantly, several psychosocial interventions have been specifically evaluated in middle-aged and older adults with schizophrenia. Cognitive Behavioral Social Skills Training (CBSST), combining cognitive behavioral therapy, social skills training, and problem-solving strategies, has been associated with improvements in social activity and overall functioning. Functional Adaptation Skills Training (FAST), which targets everyday skills such as medication management, communication, planning, transportation, and financial management, has also shown benefits in everyday functioning. In addition, integrated programs combining social rehabilitation and healthcare, such as Helping Older People Experience Success (HOPES), have demonstrated improvements in psychosocial and community functioning in older adults with severe mental illness [55]. These findings support the value of combining carefully individualized pharmacological treatment with age-adapted psychosocial interventions.

### 4.3. Pharmacological Considerations in Older Adults with Schizophrenia

Pharmacological treatment remains a cornerstone in the management of schizophrenia across the lifespan [9]. However, in older adults, medication use becomes increasingly complex due to age-related physiological changes, higher prevalence of comorbidities, and frequent polypharmacy [55]. These factors significantly influence both the efficacy and safety of antipsychotic treatment in this population.

Aging is associated with alterations in pharmacokinetics, including reduced hepatic metabolism, decreased renal clearance, and changes in body composition such as increased fat mass and reduced total body water [58]. These changes can lead to prolonged drug half-life and increased plasma concentrations, thereby raising the risk of adverse effects. In parallel, pharmacodynamic sensitivity is often enhanced in older individuals [10,59], making them more susceptible to central nervous system effects such as sedation, cognitive impairment, and extrapyramidal symptoms [60]. Particular attention should also be paid to anticholinergic burden [61], as older individuals with schizophrenia may have a history of long-term exposure to anticholinergic medications, such as biperiden, commonly used to manage extrapyramidal symptoms. Cumulative anticholinergic exposure may contribute to cognitive impairment [62] and should therefore be considered when reviewing medication regimens in this population.

Antipsychotic medications, particularly second-generation agents, are widely used in the treatment of schizophrenia but are also associated with significant side effects that may be amplified in older adults. These include metabolic disturbances, orthostatic hypotension, QT interval prolongation, and anticholinergic effects. Such adverse effects can contribute to increased risk of falls, cardiovascular events, and functional decline. Interestingly, the literature suggests that increased antipsychotic sensitivity with age comes from age-related functional decline in the dopaminergic system, including endogenous dopamine levels and dopamine receptor density [63].

Polypharmacy represents an additional challenge in this population. Older patients with schizophrenia often require treatment for multiple chronic conditions, leading to complex medication regimens and an increased risk of drug–drug interactions [46,64]. This complexity may also negatively impact treatment adherence and overall clinical outcomes.

Given these considerations, pharmacological management in older adults with schizophrenia should be individualized and carefully monitored. The principle of “start low and go slow” is commonly recommended, emphasizing cautious dose titration and regular reassessment of treatment efficacy and tolerability. In addition, periodic medication review is essential to minimize unnecessary drug exposure and reduce the risk of adverse effects. When clinically appropriate, dose reduction or discontinuation of unnecessary medications may also be considered as a strategy to reduce polypharmacy, with gradual tapering and careful monitoring to minimize the risk of withdrawal effects or symptom recurrence.

Beyond antipsychotic treatment, there is growing interest in adjunctive therapies targeting specific symptom domains or underlying biological mechanisms, such as cognitive remediation for cognitive impairment or antioxidant approaches, including N-acetylcysteine (NAC), targeting oxidative stress [52,65]. However, evidence supporting these approaches in older populations remains limited, underscoring the need for further research in this area.

## 5. Social Factors

### Psychosocial Dimensions of Aging in Schizophrenia

Beyond biological and clinical factors, psychosocial dimensions play a fundamental role in shaping the aging experience of individuals with schizophrenia. Older adults with this disorder often face a cumulative burden of social disadvantage, stigma, and reduced support networks, which can significantly impact their quality of life and overall health outcomes [48,49].

Social isolation is highly prevalent in this population and tends to increase with age. Many individuals with schizophrenia experience limited social relationships, reduced participation in community activities, and diminished access to social resources. This isolation may be further exacerbated by functional impairment, cognitive deficits, and persistent negative symptoms [66], all of which can hinder social engagement. Over time, social withdrawal may contribute not only to poorer mental health outcomes but also to increased physical morbidity and mortality [67]. Reduced social engagement may contribute to lower physical activity, poorer self-care, and reduced engagement with healthcare services, while social isolation itself has been associated with adverse physical health outcomes and increased mortality risk.

Stigma remains a major barrier to care and social inclusion [68]. Individuals with schizophrenia frequently encounter both public stigma and self-stigma [69], which can negatively affect self-esteem, treatment adherence, and willingness to seek help. In older adults, stigma may be compounded by age-related discrimination, creating a dual burden that further marginalizes this population [55].

Family and caregiver support represent critical components of care in aging individuals with schizophrenia. However, caregiving is often associated with significant emotional, physical, and economic burden [70,71]. As patients age, their caregivers—who are frequently aging relatives themselves—may face increasing challenges in providing sustained support. This dynamic underscores the importance of developing structured support systems not only for people living with schizophrenia but also for caregivers.

Socioeconomic factors, including low income, limited access to healthcare, and housing instability, further contribute to vulnerability in this population [72]. Although well-established community-based psychiatric care models are available for individuals with schizophrenia, the increasing combination of psychiatric, physical, functional, and age-related needs may pose particular challenges for older adults. Fragmentation between mental health, general medical, and geriatric services may therefore lead to gaps in care and suboptimal outcomes in this population.

Addressing these psychosocial dimensions requires a shift toward integrated, person-centered care models that prioritize social inclusion, community engagement, and multidisciplinary collaboration [73]. Interventions such as psychosocial rehabilitation, supported housing, and community-based programs may play a crucial role in improving quality of life and promoting healthy aging in individuals with schizophrenia.

## 6. Discussion

Aging in schizophrenia represents a complex and multidimensional process that cannot be fully explained by a single pathogenic mechanism [15]. Instead, it reflects the interaction between biological vulnerability, long-term disease burden, treatment-related effects, and psychosocial factors. This integrative perspective is essential to understand the heterogeneity observed in aging trajectories among individuals with schizophrenia.

From a biological standpoint, accumulating evidence supports the presence of mechanisms associated with accelerated aging, including oxidative stress, mitochondrial dysfunction, chronic inflammation, and dysregulation of stress-response systems [32,74]. These interconnected pathways may contribute to multisystem impairment, linking central nervous system alterations with peripheral comorbidities. However, the extent to which these processes are specific to schizophrenia or represent shared pathways with other chronic conditions remains an open question.

Clinically, aging individuals with schizophrenia present a high burden of physical comorbidities, persistent cognitive deficits, and increased functional dependency [55]. The overlap between long-standing cognitive impairment and potential neurodegenerative processes poses significant diagnostic challenges, particularly in older adults [10,66]. In addition, pharmacological management becomes increasingly complex due to age-related changes and the high prevalence of polypharmacy, requiring careful and individualized approaches [64].

Psychosocial factors further shape the aging experience in schizophrenia, often amplifying vulnerability. Social isolation, stigma, and caregiver burden remain critical yet frequently under-addressed aspects of care [68]. These dimensions highlight the need to move beyond a purely symptom-based approach and to consider broader determinants of health and well-being.

Despite growing interest in the intersection between aging and schizophrenia, important gaps remain in the literature. Longitudinal studies are needed to better characterize aging trajectories, identify early markers of accelerated aging, and disentangle the contributions of disease-related, treatment-related, and environmental factors. In addition, there is a need for research focused specifically on older populations, which have historically been underrepresented in clinical studies.

**Figure 1:**
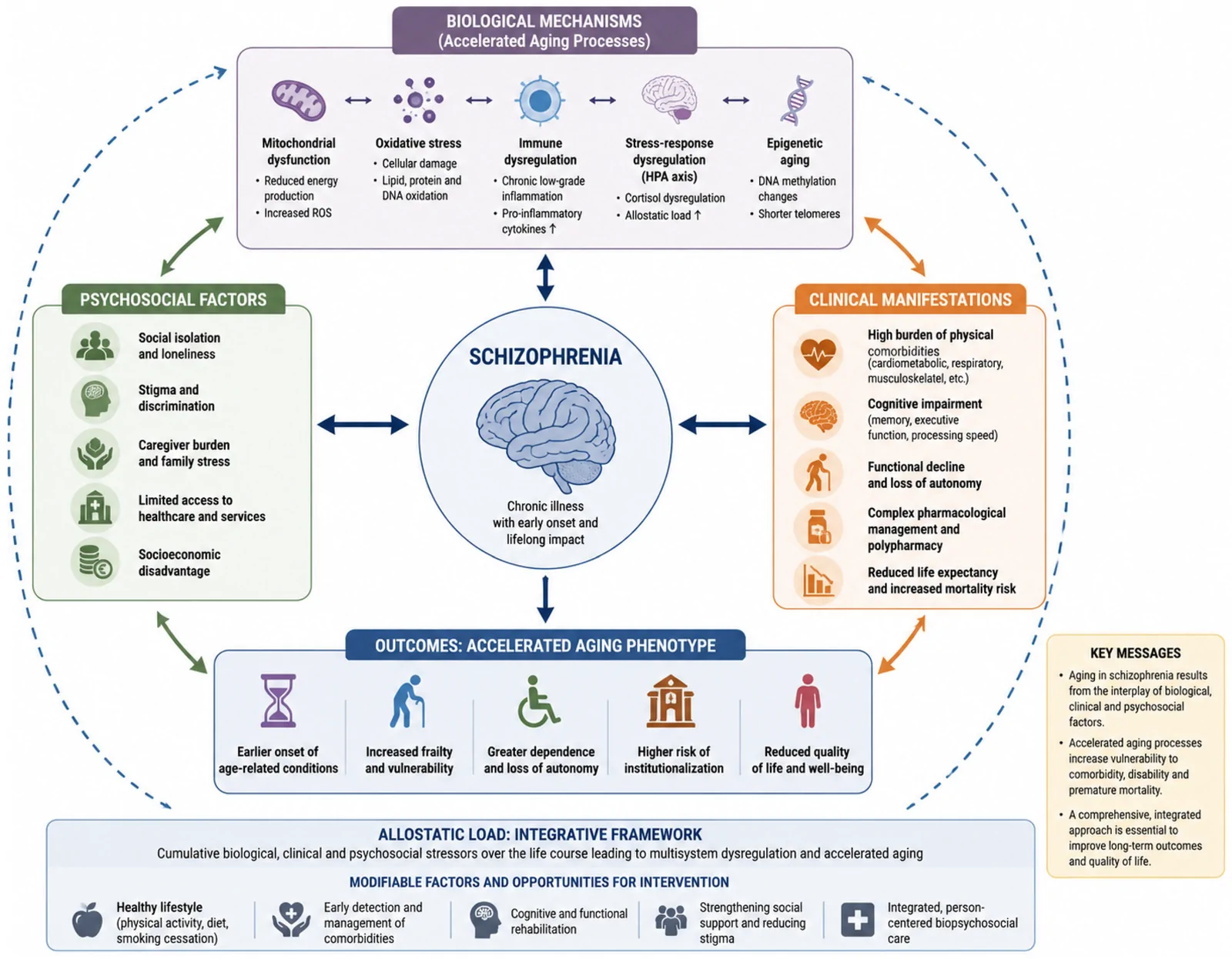
Multidimensional perspectives of aging in schizophrenia. ROS, reactive oxygen species; HPA, hypothalamic–pituitary–adrenal. Bidirectional arrows indicate reciprocal interactions between the represented factors and domains.

Overall, a more comprehensive understanding of aging in schizophrenia requires bridging biological mechanisms with clinical manifestations and lived experience [14,73]. Such an approach may contribute to the development of more effective and personalized strategies to improve long-term outcomes in this population (Figure 1). This underscores the need for integrated care models bridging psychiatry and geriatrics, along with personalized multidisciplinary strategies that incorporate pharmacotherapy and psychosocial support to mitigate functional decline and improve quality of life.

As the population with schizophrenia ages, healthcare needs increasingly extend beyond psychiatric symptom management to encompass physical multimorbidity, functional impairment, and age-related care needs. This changing clinical profile highlights the importance of developing models of care that integrate mental and general healthcare and are adapted to the specific needs of older individuals with schizophrenia [75]. Community health services may play a particularly important role in maintaining continuity of care and supporting individuals in their everyday environment, while primary healthcare is essential for the prevention, early detection, and management of the high burden of physical comorbidities in this population. Strengthening coordination between psychiatric, primary care, community, and geriatric services may therefore help reduce fragmentation and improve comprehensive care for older adults with schizophrenia. Particular attention may also be required in community and residential settings, where appropriate expertise in both psychiatric and age-related care is essential.

Notably, the number of older individuals living with schizophrenia is steadily increasing [3], reflecting both population aging and improved survival associated with advances in treatment and care. As a result, schizophrenia is progressively emerging as a condition that must be addressed not only across the lifespan but also within the context of aging-related challenges. This demographic shift underscores an urgent need to move beyond fragmented models of care and to adopt a truly integrative, biopsychosocial approach. Addressing the complex interplay between biological vulnerability, clinical burden, and psychosocial determinants is essential to respond effectively to the growing needs of this population. Ultimately, this perspective highlights not only a scientific challenge but also a pressing public health priority.

## 7. Conclusions

Aging in schizophrenia reflects a complex interaction between biological, clinical, and psychosocial factors, consistent with the concept of accelerated aging. Mitochondrial dysfunction, oxidative stress, immune dysregulation, and alterations in stress-response systems may contribute to increased biological vulnerability and the earlier emergence of age-related comorbidities. Clinically, older individuals with schizophrenia experience a substantial burden of physical comorbidity, cognitive impairment, functional decline, and polypharmacy, while psychosocial factors, including social isolation, stigma, and caregiver burden, further influence health outcomes and quality of life. The growing population of older adults with schizophrenia highlights the need for longitudinal research specifically addressing aging trajectories in this population. Ultimately, integrated, person-centered care incorporating biological, clinical, and psychosocial dimensions will be essential to address the complex needs of individuals with schizophrenia as they age.

## Data Availability

No new data were created or analyzed in this study. Data sharing is not applicable to this article.

## Abbreviations

The following abbreviations are used in this manuscript:

ATP	Adenosine triphosphate
BDNF	Brain-derived neurotrophic factor
CBSST	Cognitive Behavioral Social Skills Training
FAST	Functional Adaptation Skills Training
HOPES	Helping Older People Experience Success
HPA	Hypothalamic–pituitary–adrenal axis
MRI	Magnetic Resonance Imaging
NAC	N-acetylcysteine
OXPHOS	Oxidative phosphorylation system
PET	Positron emission tomography
ROS	Reactive oxygen species
